# Genome edited sheep generated by zygote electroporation of prime editor ribonucleoproteins

**DOI:** 10.3389/fgeed.2026.1931608

**Published:** 2026-09-22

**Authors:** Zhixuan Yu, Junjin Li, Menghao Liu, Qi Qi, Zengyi Duan, Ziyu Yang, Boyuan Guan, Jiawang Chen, Jian Hou

**Affiliations:** State Key Laboratory of Animal Biotech Breeding and College of Biological Science, China Agricultural University, Beijing, China

**Keywords:** genome editing, prime editing, ribonucleoprotein, sheep, zygote electroporation

## Abstract

Prime editing (PE) enables precise genome modifications without double-strand breaks, yet its application in large mammals has been hindered by low efficiency and the technical bottlenecks of zygote microinjection. This study aimed to establish a scalable PE platform in sheep via the direct electroporation of a PE3 RNP formulation comprising PE2 protein, an epegRNA, and a nicking sgRNA into zygotes. We first validated functional PE2 protein and screened highly efficient enhanced pegRNAs (epegRNAs) targeting two high-value traits, including short-tail (*TBXT*) and prolificacy (*FecB*), in primary sheep fibroblasts, achieving editing efficiencies of up to 22.78%. Systematic optimization of zygote electroporation determined that 60 V combined with a PE2: pegRNA mass ratio of 3.5:1 maximized delivery efficiency, yielding gene editing efficiencies of 19.8% and 20.1% for *TBXT* and *FecB*, respectively, with no statistically significant differences detected in cleavage or blastocyst rates between electroporated and untreated zygotes. Following dual-target co-delivery, intended edits were detected at both the *TBXT* and *FecB* loci in separate preimplantation embryo subsets, with locus-specific editing rates comparable to those observed under single-target conditions. Following the transfer of 54 electroporated blastocysts, 14 live lambs were born. Targeted deep sequencing detected intended edits in five lambs (35.71%), including three at the *FecB* locus and two at the *TBXT* locus. Notably, a *TBXT*-edited lamb exhibiting a distinct short-tail phenotype (15 cm vs. 24.5 cm in wild-type) carried the highest allele frequency, reaching up to 50.97%, providing direct *in vivo* functional validation. Targeted analysis of predicted off-target sites detected no credible off-target editing, supporting the specificity of this transient RNP delivery strategy. Collectively, this RNP-electroporation framework provides a feasible and precise pathway for scalable prime editing in livestock, bypassing the limitations of conventional injection-based methods.

## 1 Introduction

Since the development of the CRISPR-Cas9 system, genome editing has greatly advanced biological and agricultural research (Cong et al., 2013). However, traditional CRISPR-Cas9 relies on generating DNA double-strand breaks (DSBs), which are predominantly repaired by the error-prone non-homologous end joining (NHEJ) pathway, resulting in unpredictable insertions or deletions (indels). While homology-directed repair offers precision, it is highly inefficient in non-dividing cells, such as mammalian zygotes. Furthermore, DSBs can trigger chromosomal translocations and large deletions, raising safety concerns for embryo genome editing (Kosicki et al., 2018). In human embryos, Cas9-induced DSBs frequently remain unrepaired or are mis-repaired, leading to loss of the targeted chromosome arm or whole chromosome in a substantial proportion of embryos (Zuccaro et al., 2020; Alanis-Lobato et al., 2021). In addition to physical genomic alterations, Cas9-mediated DSBs can also activate a p53-dependent DNA damage response, leading to cell cycle arrest or apoptosis and further compromising editing safety (Haapaniemi et al., 2018).

Prime editing circumvents these limitations through a fusion of Cas9 H840A nickase and reverse transcriptase. Coupled with a prime editing guide RNA (pegRNA), it can install all 12 possible base substitutions and small insertions or deletions without requiring DSBs or exogenous DNA templates (Anzalone et al., 2019; Chen and Liu, 2023). Prime editing has been successfully applied across diverse mammalian systems, including the efficient generation of mouse models (Liu et al., 2020), and functional repair in patient-derived human organoids (Schene et al., 2020). However, translating prime editing to large livestock species has been hindered by delivery inefficiencies.

Standard delivery methods, such as viral vectors or plasmids, pose risks of random genomic integration and prolonged editor activity, which amplifies off-target effects. Microinjection remains the predominant method for delivering editing components into livestock zygotes, yet it is labor-intensive, highly dependent on operator skill, and mechanically damaging to embryos (Gim et al., 2023). Even with microinjection, prime editing efficiency in large animals remains exceptionally low. A recent study that injected prime editing mRNA into sheep zygotes to target the T-box transcription factor T (*TBXT*) and the bone morphogenetic protein receptor 1B (*BMPR1B*, also known as the *FecB* locus) p. Q249R mutation achieved only 0.2%–14.7% editing efficiencies, with no observable phenotypic changes, highlighting the intrinsic limitations of this delivery route (Zhou et al., 2023).

Delivery of pre-assembled ribonucleoprotein (RNP) complexes offers a compelling alternative by providing transient editor activity, thereby reducing off-target risks and eliminating foreign DNA integration (Kim et al., 2014; Liang et al., 2015). Recent advances have substantially expanded the feasibility of PE RNP delivery across different biological systems. Optimized lipid nanoparticle formulations have enabled *in vivo* delivery of prime editor RNPs (Hołubowicz et al., 2025), whereas cell-penetrating peptide-based strategies, such as LAH5-mediated delivery, have enhanced the intracellular uptake and editing activity of prime editor RNP complexes in mammalian cells (Yao et al., 2026). In parallel, ligation-based generation of long, chemically modified epegRNAs has improved pegRNA stability and substantially enhanced RNP-mediated PE efficiency (Lei et al., 2025). While PE RNPs have been successfully delivered via microinjection in zebrafish, providing a key proof-of-concept (Petri et al., 2022), efficient delivery of PE RNPs into large-animal zygotes via electroporation remains unexplored. Electroporation is a scalable and less invasive alternative to microinjection, and it has shown considerable promise for delivering standard Cas9 RNPs in livestock embryos (Gim et al., 2023; Fernández et al., 2024; Pi et al., 2024). However, whether electroporation can be adapted to deliver the more complex PE RNP system into large-animal zygotes remains unclear. To bridge this gap, we developed a PE3 RNP-based electroporation platform for sheep zygotes. The RNP formulation comprised PE2 protein, an epegRNA, and a nicking sgRNA. We applied this platform to loci associated with two agriculturally relevant traits. First, we targeted the short-tail phenotype naturally occurring in fat-rumped sheep, which is dictated by two linked mutations in *TBXT* (Han et al., 2019). Introducing these alleles via PE offers a genetic alternative to physical tail docking, a practice that raises significant animal welfare and hygiene concerns (Sinmez et al., 2016). Second, we targeted the A746G (Q249R) gain-of-function mutation in *FecB*, a variant extensively validated to significantly increase ovulation rate and litter size (Mulsant et al., 2001; Wilson et al., 2001). Here, we demonstrate a systematic approach to PE in a large mammal, encompassing the purification of functional PE2 protein, screening of enhanced pegRNAs, optimization of zygote electroporation parameters, and the generation of genome-edited lambs exhibiting definitive phenotypic outcomes.

## 2 Materials and methods

### 2.1 Isolation and culture of primary fibroblasts

Primary dermal fibroblasts were isolated from ear margin tissue of Mongolian sheep via enzymatic digestion. A small piece of ear tissue (5–8 mm^3^) was minced and digested in 0.25% trypsin-EDTA (Gibco, #25200056) at 37 °C for 1 h. The digestion product was filtered through a 70 μm cell strainer (Falcon, #352350), and cells were collected by centrifugation at 300 *g* for 5 min. Cells were resuspended and seeded in DMEM/F12 medium (Gibco, #11320033) supplemented with 15% fetal bovine serum (FBS, Cellmax, #SA211.02) and 1% penicillin-streptomycin (Gibco, #15140122) at 37 °C in 5% CO_2_.

### 2.2 Construction of PE2 expression plasmids and design of epegRNAs

For initial cell-based validation, the mammalian PE2 expression plasmid (pCMV-PE2) was obtained from Addgene (#132775). Enhanced pegRNAs (epegRNAs) and nicking sgRNAs targeting the ovine *TBXT* and *FecB* loci were designed using the pegFinder webtool (http://pegfinder.sidichenlab.org/) and synthesized by Sangon Biotech (Shanghai, China). All epegRNAs incorporated the engineered evopreQ1 structural motif to enhance editing efficiency (Nelson et al., 2022).

To generate templates for *in vitro* transcription (IVT), the epegRNA and nicking sgRNA expression constructs were assembled by cloning annealed oligonucleotides into linearized vectors, based on a previously described method for the PE2 system and further optimized for large-scale RNA production. The pU6-pegRNA-GG-acceptor plasmid (Addgene, #132777) and the pGL3-U6-sgRNA-PGK-puromycin vector (Addgene, #51133) served as backbones for epegRNAs and nicking sgRNAs, respectively. These vectors were linearized with BsaI-HFv2 (New England Biolabs, #R3733) and BbsI (New England Biolabs, #R3539S), respectively. Annealed oligonucleotides were ligated into the linearized vectors, and the ligation products were gel-purified to isolate the correct clones. These plasmids then served as templates for PCR amplification (primers listed in Supplementary Table S4) using Phanta Max Super-Fidelity DNA Polymerase (Vazyme, #P505-d1) to introduce the T7 promoter sequence required for subsequent transcription. The PCR products were purified using a gel extraction kit (Tiangen, #DP204) prior to use in IVT.

### 2.3 *In vitro* transcription of pegRNAs and nicking sgRNAs

The purified plasmid constructs described above served as templates for PCR amplification with Phanta Max Super-Fidelity DNA Polymerase (Vazyme, #P505-d1) using primers listed in Supplementary Table S4. This PCR step was performed to introduce a T7 promoter sequence upstream of the target region. The resulting PCR products were purified using a gel extraction kit (Tiangen, #DP204). IVT reactions were carried out with the T7 High Yield RNA Transcription Kit (Vazyme, #DD4201-01) in a 16-h incubation at 37 °C. The resulting RNA was purified by acidic phenol/chloroform extraction followed by isopropanol precipitation, and the purified pegRNAs and nicking sgRNAs were stored at −80 °C until use.

### 2.4 Prime editing validation in primary fibroblasts

Primary fibroblasts were seeded 24 h prior to transfection to reach approximately 40% confluency. Transfections were performed using Lipofectamine™ 3000 (Thermo Fisher, #L30000015) according to the manufacturer’s instructions. Each transfection mixture contained a plasmid combination of 500 ng of PE2 plasmid, 166 ng of pegRNA plasmid (Supplementary Table S1), and 55.5 ng of nicking sgRNA plasmid (Supplementary Table S2). Genomic DNA was extracted 72 h post-transfection using the TIANamp Genomic DNA Kit (Tiangen, #DP304). Target loci were amplified by PCR for 35 cycles using locus-specific primers (Supplementary Table S3) and 2× Rapid Taq Master Mix (Vazyme, #P22201). The resulting amplicons were analyzed by Sanger sequencing and subjected to deep amplicon sequencing on an Illumina NovaSeq 6000 platform (2 × 150 bp paired-end) to quantify editing efficiency.

### 2.5 Generation of PE2 protein

PE2 protein was generated based on a previously published protocol (Petri et al., 2022) with further optimizations. The PE2 coding sequence from pCMV-PE2 (Addgene, #132775) was cloned into a pET-28a (+) expression vector. To enhance soluble expression, constructs with N- or C-terminal hexahistidine (His) tags were generated. The recombinant plasmid was transformed into Rosetta (DE3) competent cells. Protein expression was induced with 0.5 mM IPTG at 20 °C for 18 h when the culture OD_600_ reached 0.7–0.8. Cells were harvested, lysed, and the clarified supernatant was subjected to Ni-NTA affinity chromatography. The eluted protein was buffer-exchanged into a low-salt storage buffer (50 mM Tris-HCl pH 7.5, 300 mM KCl, 1 mM MgCl_2_, 10% glycerol), digested with TEV protease to remove the His-tag, and passed over a second Ni-NTA column to remove the tag and protease. The resulting tag-free PE2 protein was obtained at high purity, quantified using the Bradford assay (Quick Start Bradford Protein Assay, Bio-Rad, #5000201), aliquoted, and stored at −80 °C.

### 2.6 *In vitro* nickase and reverse transcriptase activity assays

The *in vitro* nickase and reverse transcriptase activity assays were adapted from a previously described protocol (Petri et al., 2022). Sequences of all oligonucleotides are provided in Supplementary Table S5. A 51-bp double-stranded DNA (dsDNA) substrate containing a single 5′-FAM label was generated by annealing equimolar amounts of the corresponding oligonucleotides (10 µM each) at 95 °C for 3 min, followed by slow cooling to room temperature; a nicking event on this substrate yields a 5′-FAM-labeled product of 34 nt. To assess the nickase activity of the purified PE2 protein, RNP complexes were assembled by incubating the purified PE2 protein with *in vitro*-transcribed pegRNA at room temperature for 10 min. The nicking reaction was initiated by adding 1 µL of 10× reaction buffer, 1 µL of the annealed FAM-labeled dsDNA substrate (2 μM, 2 pmol), and RNase-free water to a final volume of 10 µL. The components were mixed at a molar ratio of 1:10:16 (dsDNA:PE2:pegRNA), consistent with established protocols (Petri et al., 2022). The mixture was incubated at 37 °C for 4 h to ensure complete substrate turnover. Negative controls (no RNP) and positive controls (Cas9 RNP) were included in each run to exclude non-specific cleavage. Reactions were terminated by adding an equal volume of 2× urea loading buffer, heating at 95 °C for 5 min, and immediately quenching on ice for 3 min. Samples were resolved on a heated 15% denaturing polyacrylamide gel containing 8 M urea in TBE buffer at 150 V for 70 min. FAM-labeled DNA bands were visualized by laser excitation at 480 nm. Nicking efficiency was quantified by densitometry as the ratio of the cleaved product intensity to the total lane intensity.

To evaluate the reverse transcriptase activity, the nicking reaction described above was subsequently used. Following the 4-h nicking incubation, 1 µL of dNTP mix (10 mM each) was added directly to the reaction product. The mixture was gently vortexed and incubated at 37 °C for an additional 4 h to allow reverse transcription. The reaction was terminated and analyzed using the same urea-PAGE procedure as described for the nicking assay. Extension products derived from the FAM-labeled nicked strand were detected by laser excitation at 480 nm.

### 2.7 Oocyte collection, *in vitro* maturation, and fertilization

Ovaries from sexually mature ewes were obtained from a local abattoir and transported to the laboratory within 2 h in sterile physiological saline (0.9% NaCl) at 37 °C. Cumulus-oocyte complexes (COCs) were aspirated from 3 to 6 mm follicles in diameter using a disposable syringe. COCs with homogeneous cytoplasm and multiple compact layers of cumulus cells were selected for *in vitro* maturation (IVM). The COCs were cultured in IVM medium, TCM-199 (Sigma-Aldrich), supplemented with 10% estrus sheep serum, 10 μg/mL FSH (Follitropin-V; Bioniche Inc., Belleville, Ontario, Canada), 10 μg/mL LH (Bioniche Inc.), 1 μg/mL 17-β estradiol (Sigma-Aldrich), and 1% penicillin-streptomycin (Sigma-Aldrich). Culture was carried out for 22–26 h at 38.5 °C under 5% CO_2_ in a humidified atmosphere. Following IVM, the surrounding cumulus cells were removed by treatment of COCs with 2% hyaluronidase, and the denuded oocytes were placed in fertilization medium, synthetic oviduct fluid (SOF) supplemented with 2% estrus sheep serum. For *in vitro* fertilization (IVF), frozen–thawed semen was processed for sperm capacitation using the swim up method as described previously (Qi et al., 2025). Capacitated sperm were adjusted to a final concentration of 1 × 10^6^ sperm/mL in fertilization medium and co-incubated with oocytes for 6 h to achieve fertilization.

### 2.8 Preparation of RNP complexes and electroporation of zygotes

To prepare the PE3 RNP formulation, purified PE2 protein (20 μL at 3.5 μg/μL; 70 μg), epegRNA (2 μL in total at 10 μg/μL; 20 μg), and nicking sgRNA (0.4 μL in total at 10 μg/μL; 4 μg) were mixed and incubated at room temperature for 10–15 min to allow RNP assembly. Opti-MEM was then added to obtain a final electroporation volume of 40 μL. The final mass concentrations of PE2 protein, total epegRNA, and total nicking sgRNA were 1.75, 0.50, and 0.10 mg/mL, respectively, corresponding to a mass ratio of 3.5:1:0.2. Based on an estimated molecular mass of 260 kDa for PE2 and RNA lengths of approximately 160–180 nt for the epegRNAs and 96 nt for the nicking sgRNAs, the corresponding molar concentrations were approximately 6.7 μM PE2, 8.2–9.2 μM total epegRNA, and 3.1 μM total nicking sgRNA. Thus, the approximate PE2: total epegRNA: total nicking sgRNA molar ratio was 1:1.3:0.46. The exact molar ratio varied slightly among target-specific RNP formulations because of differences in epegRNA length.

Sheep zygotes 6 h after IVF were washed with *in vitro* culture (IVC) medium, SOF supplemented with 8 mg/mL bovine serum albumin (BSA; Sigma-Aldrich), 1% (v/v) essential amino acids, and 2% (v/v) non-essential amino acids (Sigma-Aldrich). The washed zygotes were placed in fresh 50-µL droplets of IVC medium and briefly held at 38.5 °C under 5% CO_2_ prior to electroporation. For electroporation, batches of 50–60 zygotes were rinsed in pre-chilled Opti-MEM (total handling time <120 s) and transferred to a 2 mm gap cuvette (BTX, 45–0135) containing 40 µL of the RNP electroporation solution. Electroporation was performed using a BTX ECM™ 830 square wave generator. During voltage optimization, 50, 60, and 70 V were tested using a fixed pulse program consisting of three unipolar square-wave pulses, each with a duration of 3 ms and an interval of 100 ms. Based on embryo viability and delivery efficiency, 60 V, corresponding to an electric field strength of 300 V/cm in a 2-mm-gap cuvette, was selected and used for all subsequent electroporation experiments. Immediately after pulsing, embryos were examined under a stereomicroscope. Those with normal morphology were washed and cultured in IVC medium at 38.5 °C in 90% N_2_, 5% CO_2_, 5% O_2_. Cleavage and blastocyst formation were recorded at 48 h and 6–8 days after IVF, respectively.

### 2.9 Genotyping of preimplantation embryos

On day 7 after IVF, individual embryos ranging from the 4-cell stage to the blastocyst stage were collected. Each embryo was transferred into 10 μL of ddH_2_O and lysed by two freeze-thaw cycles, each consisting of freezing at −20 °C for 30 min and thawing at room temperature for 10 min. The entire lysate was used as the template for the first-round PCR, which was performed for 35 cycles using the corresponding outer primer pair (F1/R1; Supplementary Table S3) and 2× Rapid Taq Master Mix (Vazyme, P222-01). Subsequently, 0.2 μL of the first-round product was subjected to a second round of 35-cycle PCR using the corresponding inner primer pair (F2/R2; Supplementary Table S3) and the same master mix.

The second-round products were examined on a 1% agarose gel. Products showing a single band of the expected size without detectable nonspecific amplification were submitted to Sangon Biotech (Shanghai, China) for Sanger sequencing. Sequencing chromatograms were visualized and aligned to the corresponding wild-type reference sequences using SnapGene software (version 4.3.6). An embryo was classified as edited when the intended nucleotide substitution was detected at the expected target position and was absent from the wild-type control. The embryo-level editing rate was calculated as the number of successfully sequenced embryos carrying the intended edit divided by the total number of successfully sequenced embryos and expressed as a percentage.

Because individual embryos yielded limited amounts of genomic DNA and whole-genome amplification was not performed, each embryo was analyzed at either the *TBXT* or *FecB* locus, but not both. The two loci in the dual-target co-delivery group were therefore evaluated in separate, non-overlapping embryo subsets.

### 2.10 Production of gene-edited lambs

All animal experimental procedures were conducted in accordance with the animal care and welfare guidelines of China Agricultural University and approved by its Animal Ethics Committee (Protocol code: AW70405202-3–03, Approval Date: 7 April 2025). All animals were housed at the farm of Inner Mongolia Sino Sheep Technology Co., Ltd.

Suffolk donor ewes (2–3 years old) were synchronized using an intravaginal progesterone-releasing device (CIDR, 0.3 g; InterAg, New Zealand) for 13 days. Subsequently, ewes were superovulated using a decreasing regimen of follicle-stimulating hormone (FSH), with a total dose of 420 IU administered over six injections at 12-h intervals (Ningbo Sansheng Pharmaceutical, China). Approximately 12 h after the last FSH injection, COCs were collected via laparoscopic follicular aspiration under general anesthesia. The COCs with uniform cytoplasm and intact cumulus layers were selected for IVM and IVF. The derived zygotes were electroporated with PE RNPs and cultured up to blastocysts, as the procedure described above. Well-developed blastocysts were transferred into the uteri of synchronized recipient ewes, with 1–2 embryos per recipient. Pregnancy was diagnosed by B-mode ultrasonography at 45 ± 2 days post-transfer, and lambs were delivered at term (∼150 days) with standard neonatal care.

### 2.11 Genotyping and off-target assessment

Genomic DNA was extracted from peripheral blood samples collected by jugular venipuncture from lambs at approximately 10 days of age. To genotype the edited animals, target regions of the ovine *TBXT* and *FecB* genes were amplified by PCR using specific primers (Supplementary Table S3) and a high-fidelity DNA polymerase. The PCR products were analyzed by Sanger sequencing for preliminary genotyping and by targeted deep sequencing for quantitative analysis. For targeted deep sequencing, the amplicons were purified using magnetic beads and used to construct adapter-indexed libraries. Following library purification and quality assessment, sequencing was performed by Suzhou Junji Biotechnology Co., Ltd (Suzhou, China) on an Illumina NovaSeq 6,000 platform using v1.5 sequencing reagents with a 2 × 150-bp paired-end configuration. Reads passing the service provider’s quality-control pipeline were used for analysis, and the variant allele frequency (VAF) was calculated as the number of reads carrying the intended substitution divided by the total number of reads covering the target nucleotide.

Potential off-target sites for the pegRNAs and nicking sgRNAs were predicted *in silico* using CRISPOR (Concordet and Haeussler, 2018), allowing for up to three mismatches and requiring an NGG protospacer adjacent motif (PAM). Specific primers were designed to amplify the predicted off-target loci (five for *TBXT* and seven for *FecB*; off-target sites are listed in Supplementary Table S6, and primer sequences in Supplementary Table S7). The resulting amplicons were subjected to Oxford Nanopore sequencing to comprehensively assess the presence of off-target editing.

### 2.12 Statistical analysis

All graphs were generated using GraphPad Prism software (version 8.0.3). Data presented in bar and line graphs are expressed as mean ± standard error of the mean (SEM) from three independent biological replicates. Statistical significance was evaluated using one-way analysis of variance (ANOVA) and Student’s t-test. Differences were considered significant at *P* < 0.05, *P* < 0.01.

## 3 Results

### 3.1 Expression and purification of functional PE2 protein

We sought to produce highly purified and active PE2 protein. To achieve high soluble expression in *E. coli*, we employed an optimized construct with a C-terminal 10×His tag (pET-PE2-10His). Following nickel affinity chromatography and TEV protease-mediated tag removal, we obtained tag-free PE2 protein at high purity (Figure 1A). All subsequent assays used this preparation.

**FIGURE 1:**
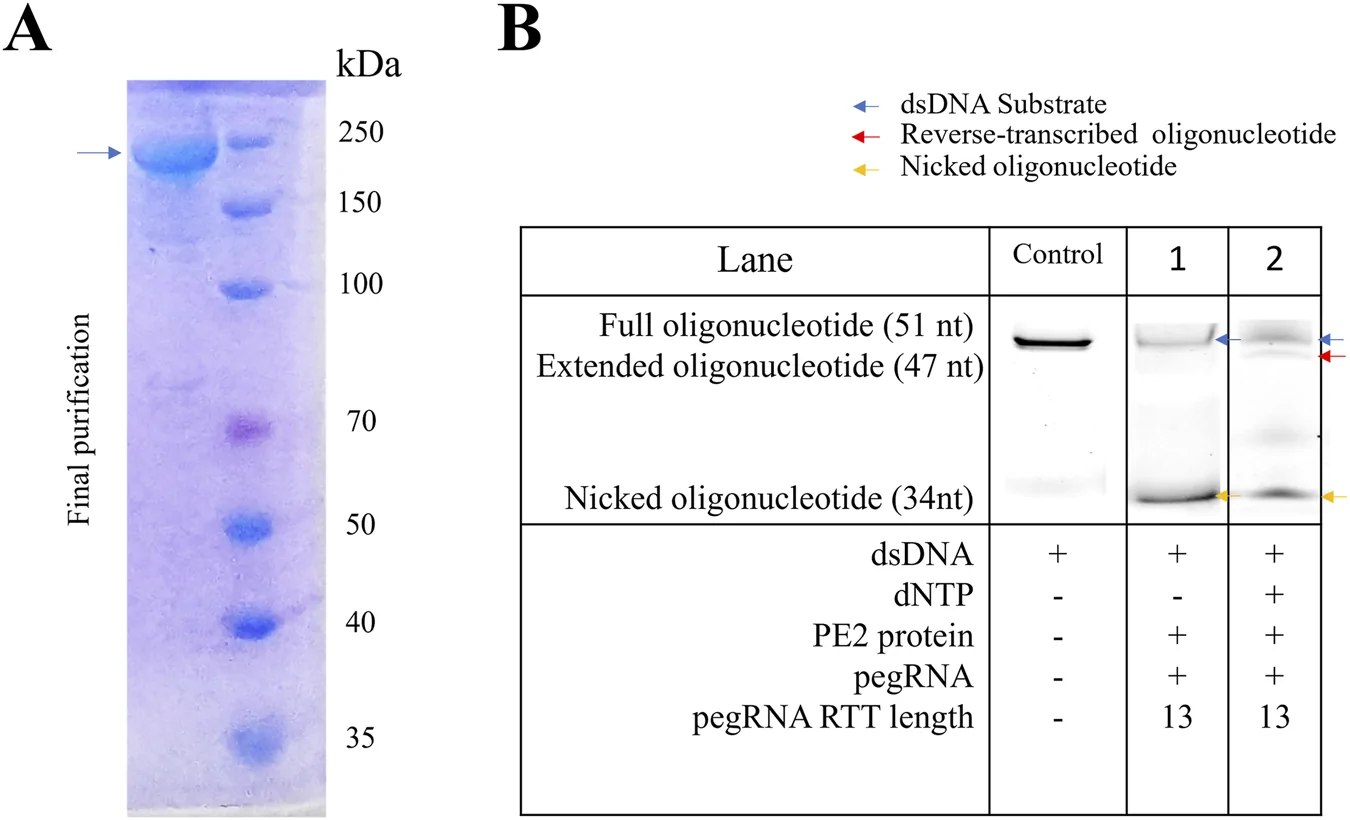
Purification and *in vitro* functional validation of PE2 protein. **(A)** SDS-PAGE analysis of purified, tag-free PE2 protein expressed from the pET-PE2-10His vector. The protein was purified via nickel affinity chromatography, buffer exchange by ultrafiltration, and tag removal by TEV protease. The blue arrow indicates the PE2 protein band. **(B)** *In vitro* activity assay demonstrating the nickase and reverse transcriptase activities of purified PE2 protein. Lanes 1 and 2 were obtained from separately performed reactions under different conditions. Lane 1 contained PE2 protein and pegRNA without dNTPs, whereas lane 2 contained PE2 protein, pegRNA, and dNTPs. The blue, yellow, and red arrows indicate the 51-nt substrate, 34-nt nicked product, and 47-nt extension product, respectively. The uncropped source image and complete reaction conditions are provided in Supplementary Figure S1B.

We next verified that the purified PE2 protein retained both nickase and reverse transcriptase activities *in vitro*. This was assessed using a fluorescently labeled dsDNA nicking assay and a subsequent in-solution primer extension assay. Assembled into an RNP with a guide RNA, PE2 cleaved a fluorescent 51-nt DNA substrate into the expected 34-nt fragment (Figure 1B; Supplementary Figure S1A). When dNTPs and a pegRNA template were added to the reaction, a distinct 47-nt extension product appeared (Figure 1B, lane 2; Supplementary Figure S1B), confirming robust reverse transcriptase activity. Thus, the purified protein possesses the dual enzymatic activities required for prime editing.

### 3.2 Identification of efficient pegRNAs in sheep primary fibroblasts

To identify the most efficient pegRNAs for embryo editing, we first screened candidates in sheep primary fibroblasts. We used a plasmid-based PE3 system (PE2 expression plasmid, pegRNA, and nicking sgRNA) for this initial step, as it allows rapid, parallel evaluation of multiple pegRNA designs without consuming large amounts of purified PE2 protein. Sanger sequencing confirmed that the PE3 system mediated editing at both target loci (Figures 2A,C). We then used targeted deep sequencing to accurately quantify this editing (primers in Supplementary Table S3).

**FIGURE 2:**
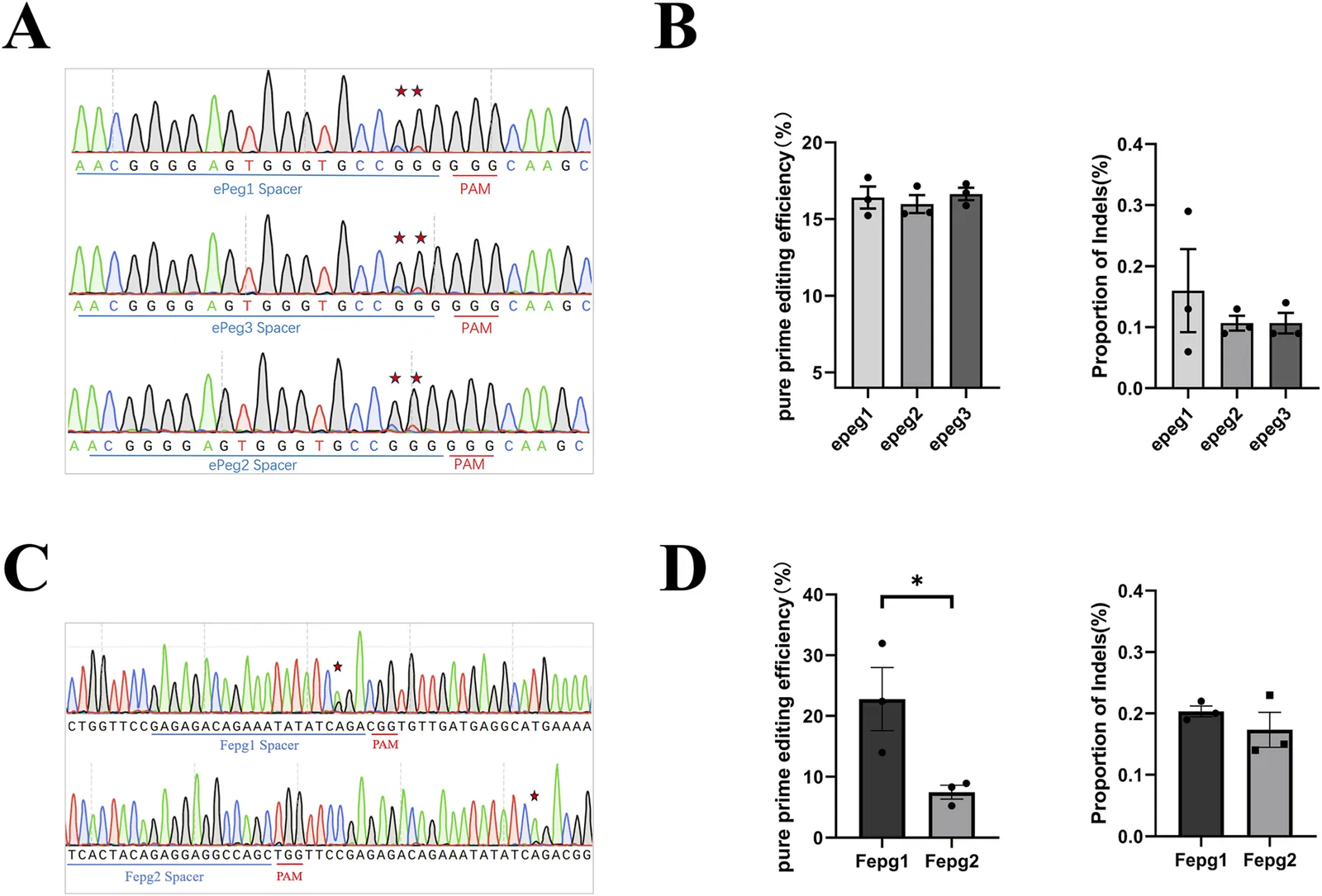
Validation of prime editing in primary sheep fibroblasts. **(A)** Representative Sanger sequencing chromatograms confirming edits at the *TBXT* locus. The spacer (blue underline), PAM (red underline), and target base (asterisk) are indicated. **(B)** Precise editing efficiency (left bars) and corresponding indel frequency (right bars) for three *TBXT*-targeting epegRNAs (epeg1–epeg3), quantified by amplicon deep sequencing (mean ± SEM, n = 3). **(C)** Representative Sanger sequencing chromatograms confirming edits at the *FecB* locus. Sequence annotations as in **(A). (D)** Precise editing efficiency (left bars) and corresponding indel frequency (right bars) for two *FecB*-targeting epegRNAs (Fepg1–Fepg2), quantified by amplicon deep sequencing (mean ± SEM, n = 3).

For *TBXT*, we screened three epegRNAs, designated epeg1–epeg3, based on the engineered pegRNA design (Nelson et al., 2022). Epeg3 achieved the highest precise editing efficiency of 17.72%, with a minimal indel background (approximately 0.10%; Figure 2B). For *FecB*, we evaluated two candidates (Fepg1–Fepg2). Fepg1 mediated the A746G (Q249R) substitution with an efficiency of 22.78%, substantially higher than Fepg2, while maintaining a low indel frequency (0.2%; Figure 2D). Therefore, the epeg3 and Fepg1 were selected for embryo experiments. Primers for *in vitro* transcription are listed in Supplementary Table S4.

### 3.3 Optimization of PE3 RNP electroporation in sheep zygotes

The overall workflow for generating gene-edited sheep by PE3 RNP electroporation is illustrated in Figure 3A. We used *in vitro* fertilized zygotes produced from slaughterhouse ovary-derived oocytes to establish an electroporation protocol. First, we optimized the voltage. While 50 V and 60 V did not significantly affect cleavage or blastocyst rates compared to the control (P > 0.05), 70 V caused severe cytoplasmic leakage (Supplementary Figure S2A) and significantly reduced embryo survival (P < 0.05). We selected 60 V as the standard parameter to balance delivery efficiency and embryo viability (Figure 3B; Supplementary Table S8).

**FIGURE 3:**
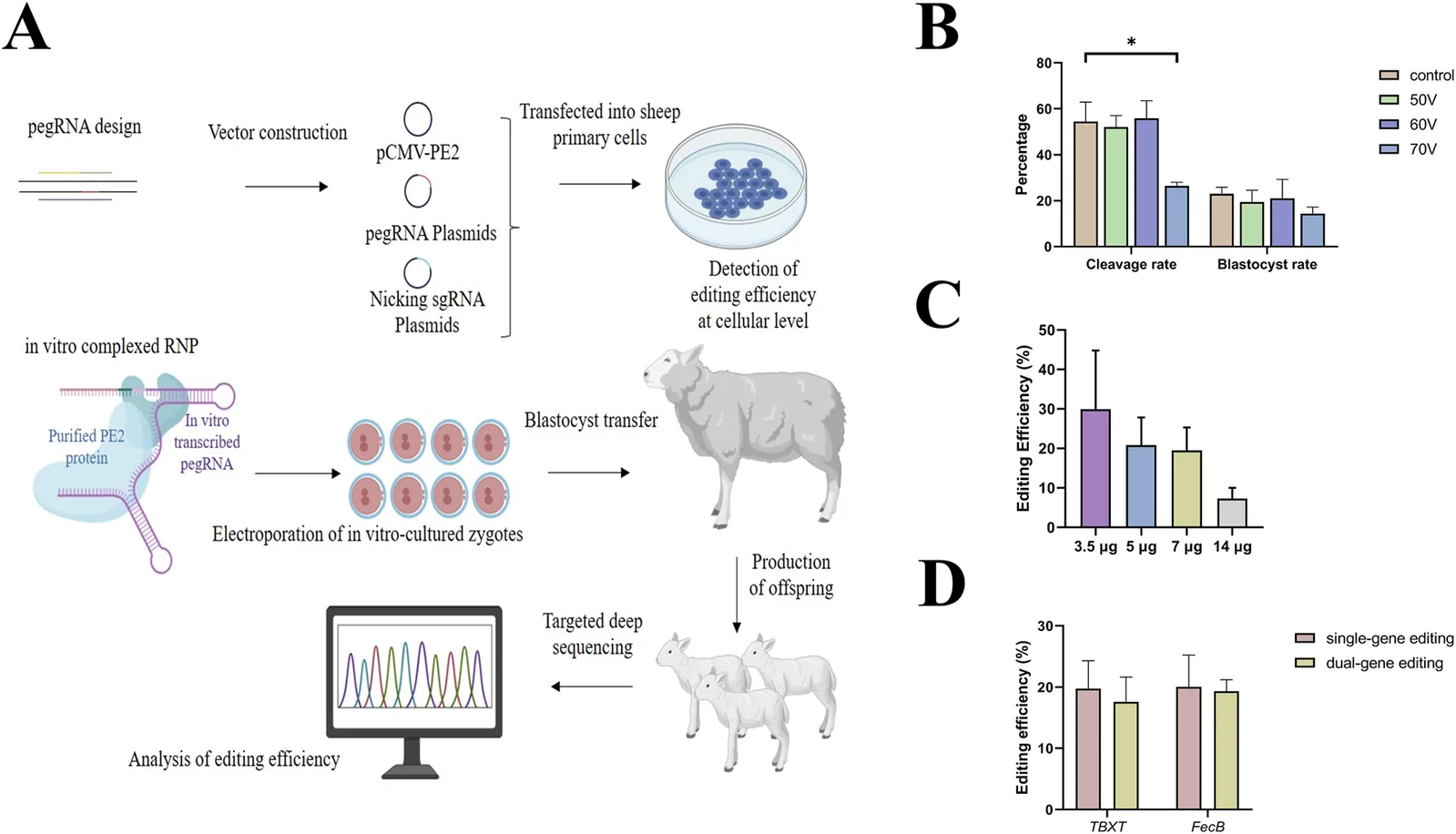
Optimization of the PE3 RNP electroporation system in sheep embryos. **(A)** Schematic workflow for generating gene-edited sheep via PE3 RNP electroporation. **(B)** Optimization of electroporation voltage. Embryo development was compromised at 70 V (P < 0.05), while 50 V and 60 V showed no significant adverse effects on cleavage rate and blastocyst formation (mean ± SEM, n = 3). **(C)** Optimization of PE2:pegRNA mass ratio. Editing efficiency was tested with PE2 protein amounts of 3.5 μg, 5 μg, 7 μg, and 14 μg per 1 μg of pegRNA. A 3.5:1 ratio yielded the highest editing efficiency in blastocysts (mean ± SEM, n = 3). **(D)** Locus-specific editing following dual-target co-delivery. Editing rates at *TBXT* and *FecB* were determined in separate embryo subsets and compared with those obtained under single-target conditions (mean ± SEM, n = 3).

Next, using the *TBXT* locus as a model, we evaluated four PE2: epegRNA mass ratios: 3.5:1, 5:1, 7:1, and 14:1. Ratios below 3.5:1 were not examined in the present study. Among the conditions tested, the 3.5:1 ratio produced the highest editing efficiency (29.9%; Figure 3C; Supplementary Table S9). We therefore selected a PE2: epegRNA: nicking sgRNA mass ratio of 3.5:1:0.2 for the subsequent experiments. With these optimized parameters, we co-delivered PE3 RNPs targeting *TBXT* (epeg3) and *FecB* (Fepg1). The recovered preimplantation embryos were divided into separate, non-overlapping subsets and genotyped at either the *TBXT* or *FecB* locus by nested PCR and Sanger sequencing. Intended edits were detected at both loci, with locus-specific editing rates comparable to those observed under single-target conditions (Figure 3D; Supplementary Table S10). These findings demonstrate that both target-specific PE3 RNPs retained editing activity under dual-target co-delivery conditions, although co-editing of the two loci within individual embryos was not assessed.

### 3.4 Generation of gene-edited lambs by PE3 RNP electroporation

To evaluate preimplantation development under the optimized electroporation conditions, oocytes collected from FSH-superstimulated Suffolk donor ewes were matured and fertilized *in vitro*. Across five independent sessions, 212 untreated zygotes from the same donor pool were assigned to the control group, whereas 198 zygotes underwent PE3 RNP electroporation. No statistically significant differences in cleavage or blastocyst rates were detected between the two groups (Figure 4A; Supplementary Table S11).

**FIGURE 4:**
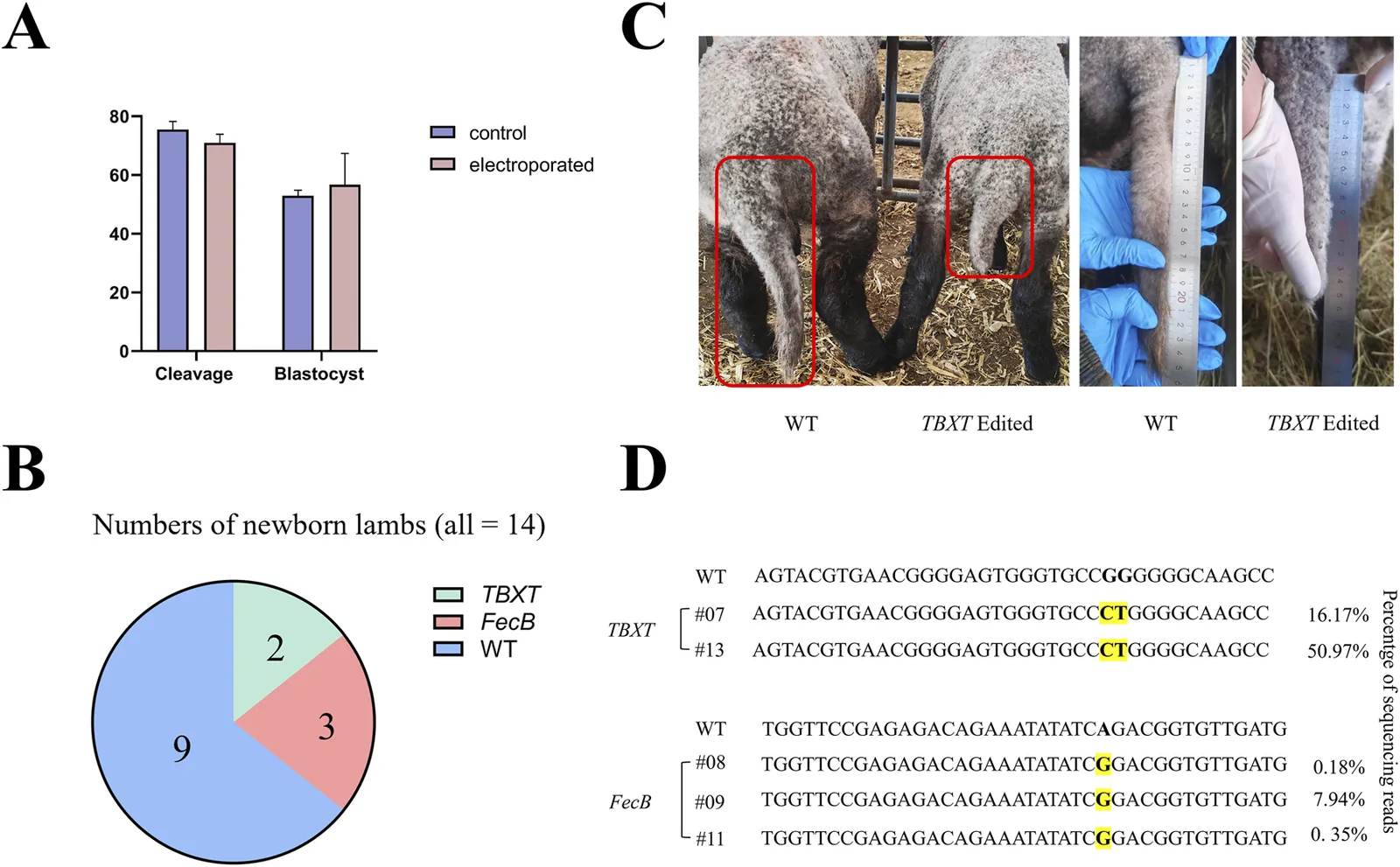
Generation of phenotypically validated gene-edited lambs. **(A)** Preimplantation development of zygotes from live donor sheep. No statistically significant differences in cleavage or blastocyst rates were detected between PE3 RNP-electroporated and unelectroporated control zygotes (mean ± SEM, n = 5). **(B)** Genotyping summary of live-born lambs (n = 14). Five lambs carried precise edits: two at *TBXT* and three at *FecB*. **(C)** Phenotypic validation. A *TBXT*-edited lamb (#13) exhibits a shortened tail compared to a wild-type control. **(D)** Representative deep sequencing sequence alignments confirming precise edits in live lambs. The target base is highlighted in yellow.

A separate cohort was used for live-animal production. A total of 203 oocytes were collected from 14 donor ewes and subjected to IVM and IVF. The resulting pronuclear-stage zygotes were electroporated with PE3 RNPs and cultured, yielding 123 cleaved embryos and 84 blastocysts. Of these, 54 top-quality blastocysts were transferred into 33 synchronized recipient ewes (Table 1; Supplementary Figure S2B). Ultrasound examination on day 45 identified 18 pregnant recipients (54.55%), resulting in the birth of 14 live lambs. Targeted deep sequencing identified five edited individuals: three carrying the *FecB* edit and two carrying the *TBXT* edit (Figure 4B; Table 1). Across these five lambs, the observed VAFs for the intended substitutions ranged from 0.18% to 50.97% in genomic DNA extracted from peripheral blood (Figure 4D; Supplementary Figure S3), suggesting mosaic editing in some lambs. The two low-frequency *FecB* calls in lambs #08 and #11 were supported by large numbers of strand-balanced sequencing reads. Owing to the absence of independent technical replicates and a formally defined limit of detection, these two events were regarded as provisional. Overall, the deep-sequencing-based detection rate was 35.71% (5/14), with locus-specific rates of 21.43% (3/14) for *FecB* and 14.29% (2/14) for *TBXT.* These estimates include the two provisional *FecB* calls.

**TABLE 1:** Summary of embryo transfer and gene-editing efficiency in live lambs.

Parameter	Number (%)
Donors	14
Collected oocytes	203
Cleavage number	123 (60.59%; 123/203)
Blastocyst number	84 (68.29%; 84/123)
Transplanted blastocysts	54
Transplanted recipients	33
Pregnancy recipients	18
No. of lambs born	14
No. of *FecB*-edited lambs	3
No. of *TBXT*-edited lambs	2

Most notably, lamb #13 carried the two intended linked *TBXT* substitutions (c.333G>C and c.334G>T), recreating the naturally occurring short-tail-associated haplotype. The edited haplotype was detected at a frequency of 50.97% in peripheral blood-derived DNA, compatible with a heterozygous editing pattern. This lamb exhibited a distinct short-tail phenotype (15 cm versus 24.50 ± 2.54 cm in wild-type controls; Figure 4C; Supplementary Table S12).

To assess off-target editing, we used CRISPOR to predict potential off-target sites (CFD >0.1, ≤3 mismatches) for both pegRNAs. We then amplified the five top-ranked sites for *TBXT* and seven for *FecB* from the edited lambs’ genomic DNA for Nanopore sequencing. All examined sites showed no detectable sequence changes relative to the wild-type controls (Supplementary Figure S4). Thus, no credible off-target editing was detected at the CRISPOR-predicted candidate sites examined.

## 4 Discussion

The establishment of an efficient prime editing (PE) platform for large livestock species represents a critical step toward translating genome editing technologies into practical animal breeding. In this study, we developed a RNP-based electroporation protocol for delivering a PE3 RNP formulation comprising PE2 protein, an epegRNA, and a nicking sgRNA into sheep zygotes, achieving intended edits at the *TBXT* and *FecB* loci. In this configuration, the nicking sgRNA directs a second nick in the non-edited strand and can favor repair toward the edited sequence, thereby improving PE efficiency relative to PE2 (Anzalone et al., 2019). However, because a PE2-only RNP control was not included in the embryo experiments, the specific contribution of the nicking sgRNA to editing efficiency and indel formation could not be determined. Although low indel frequencies were observed for the selected guide combinations in the fibroblast screen, their effects on indel formation in embryos were not directly assessed. Notably, one *TBXT*-edited lamb exhibited a clear short-tail phenotype, providing functional validation of our approach.

Previous studies have demonstrated that Cas9 RNPs could be delivered efficiently into sheep zygotes by electroporation (Mahdi et al., 2022). Those authors report optimized electroporation parameters involving four 40–42 V pulses of 3.5 ms at 50 ms intervals using a cuvette with a 1 mm electrode gap. This protocol achieved high rates of biallelic knockout in blastocysts and was subsequently used to generate lambs carrying SOCS2 mutations (Mahdi et al., 2025). Due to the limited availability of sheep zygotes, we kept the pulse number (3 pulses), duration (3 m), and interval (100 m) constant during the initial screen and focused optimization on voltage. We suggest that 60 V would be suitable for balancing delivery efficiency and embryo viability. As we used a cuvette with a 2 mm electrode gap, the nominal electric field strength was lower in our system than in the previous protocol (300 versus 400 V/cm). In addition to the voltage, the PE2:epegRNA ratio was also screened in this study, and 3.5:1 was regarded as the best-performing ratio among the conditions tested. Ratios below 3.5:1 were not evaluated, because the 3.5:1 mass ratio already represents a slight molar excess of epegRNA relative to PE2, and further increasing the RNA excess would affect RNP assembly, intracellular component balance, and editing activity. Nevertheless, further optimization of the electroporation-based program may improve PE3 delivery efficiency and gene editing outcomes. We currently conclude that although knockout with Cas9 and sequence conversion with PE3 involve different mechanisms and editing endpoints, both approaches support electroporation as a practical method for RNP delivery into sheep zygotes.

A key advantage of our platform lies in the editing depth and functional outcomes it achieves. In our study, the targeted allele mutation frequencies in the *TBXT*-edited lambs reached 16.17% and 50.97%, and those in the *FecB*-edited lambs ranged from 0.18% to 7.94%. This contrasts sharply with the prior study (Zhou et al., 2023), in which microinjection of PE mRNA targeting the same ovine loci yielded substantially lower allele frequencies (0.2%–14.7% for *TBXT* and 0.18%–1.31% for *FecB*) and produced no observable phenotypic changes. We attribute this improved editing depth and phenotypic penetrance to both the precise developmental timing and the mechanistic benefits of RNP delivery. In the previous study, embryos derived from *in vivo* fertilization were microinjected at uncertain developmental stages due to the unpredictable timing of mating (Zhou et al., 2023). In contrast, our IVF-based platform allows electroporation at a strictly defined time point (6 h post-fertilization), ensuring that editing occurs during a narrow, synchronous window of zygotic development. Concurrently, pre-assembled PE3 RNPs are immediately active upon entry, providing a transient burst of high-concentration editing activity that bypasses the transcriptional and translational delays inherent to mRNA delivery. This rapid pulse of activity, followed by protein degradation, also minimizes off-target risks. Furthermore, electroporation provides critical practical advantages over conventional microinjection, allowing the simultaneous co-delivery of two pegRNAs to simplify the workflow and reduce embryo use, while also enabling the parallel processing of multiple zygotes for the scalability essential in agricultural applications.

The precision of our approach distinguishes it from DSB-dependent editing strategies, although it also highlights a trade-off between precision and efficiency. For *TBXT*, a recent HDR-based study targeting *TBXT* in sheep also produced tail shortening, but the most pronounced phenotype was associated with an unintended 8-bp deletion alongside the desired substitution (Li et al., 2022). In contrast, the short-tail lamb in our study carried a clean, defined allele, highlighting the capacity of prime editing to avoid the collateral indels that frequently accompany DSB repair. However, for *FecB*, where CRISPR/Cas9-mediated HDR has proven highly efficient (Zhang et al., 2025), our PE approach yielded substantially lower allele frequencies. In our study, blood-derived VAFs were lower at the *FecB* locus than at the *TBXT* locus, which may partly reflect locus-specific sequence features affecting prime editing activity (Mathis et al., 2023). As these measurements were limited to peripheral blood, multi-tissue genotyping would help characterize editing patterns across tissues.

Our RNP electroporation platform is well suited to incorporate advances in prime editing technology. Recent work has shown that next-generation PE systems, such as uPEn delivered as mRNA, achieve substantially higher knock-in efficiencies in mouse and sheep zygotes (Mao et al., 2025). Adapting these enhanced editors to our RNP electroporation framework could combine their superior catalytic activity with the safety and scalability inherent to RNP delivery, potentially enabling more complex edits in livestock embryos. No off-target variants were detected at the CRISPOR-predicted candidate sites examined in peripheral blood-derived genomic DNA, supporting a low level of off-target activity at these sites. Nevertheless, the targeted assays used here cannot exclude low-frequency mosaic variants or editing at unpredicted loci, and overall Nanopore read identity does not replace locus-specific variant analysis. More sensitive amplicon sequencing and unbiased genome-wide approaches, such as PEAC-seq, CIRCLE-seq, or whole-genome sequencing, will be valuable for further evaluating the genomic safety of this platform (Yu et al., 2022; Inen et al., 2024).

In conclusion, we have developed a practical and scalable PE RNP electroporation platform for precise genome editing in sheep. By optimizing the delivery parameters of the PE3 RNP formulation, we achieved editing at two agriculturally relevant loci, detected locus-specific editing at both targets following dual-target co-delivery in separate preimplantation embryo subsets, and generated live lambs carrying precise single-locus edits, including a *TBXT*-edited lamb with a defined short-tail phenotype. This platform provides a flexible foundation for deploying future prime editing tools in livestock and represents a concrete step toward integrating precision genome editing into animal breeding programs.

## Data Availability

The original contributions presented in the study are included in the article/Supplementary Material, further inquiries can be directed to the corresponding author.
