# Supplementary material for: Genome edited sheep generated by zygote electroporation of prime editor ribonucleoproteins
Source: Front Genome Ed. 2026 Sep 22;8:1931608. doi: 10.3389/fgeed.2026.1931608 (PMC13638620; doi:10.3389/fgeed.2026.1931608)
Supplement: Supplementary file 1 [file Supplementaryfile1.docx]

**Genome Edited Sheep Generated by Zygote Electroporation of Prime Editor Ribonucleoproteins**

Zhixuan Yu ^1^, Junjin Li ^1^, Menghao Liu ^1^, Qi Qi ^1^, Zengyi Duan ^1^, Ziyu Yang ^1^, Boyuan Guan ^1^, Jiawang Chen ^1^, Jian Hou ^1*^

State Key Laboratory of Animal Biotech Breeding and College of Biological Science, China Agricultural University. Beijing, China.

*** Corresponding author.**

E-mail address: [houjian@cau.edu.cn](mailto:houjian@cau.edu.cn)

**Supplementary material**

Supplementary Figure S1～4.

Supplementary Table S1～12

**Supplementary Figures**

**Figure S1. *In vitro* validation of PE2 RNP activity.**

**(A)** Nicking activity of PE2 RNP. A 51-bp fluorescently labeled dsDNA substrate was used. When PE2 protein was assembled with either sgRNA or pegRNA into RNP complexes and incubated, efficient cleavage occurred at the specific site of the dsDNA substrate, generating a 34-nt single-stranded DNA (ssDNA) fragment, as confirmed by denaturing PAGE analysis.

(B) Reverse transcription activity of PE2 RNP. Building on the nicking assay, dNTP mix was added to the reaction system containing PE2-pegRNA RNP. In addition to the 34-nt cleavage product, two new DNA bands of approximately 46 nt and 47 nt (indicated by blue and green arrows) were clearly observed. These bands correspond to the theoretical values: reverse transcription extension of 12 nt or 13 nt on the 3’ overhang generated by nicking, using the RTT sequence of pegRNA as a template.

**Figure S2. Effect of electroporation voltage on sheep zygotes. (A)** Representative bright-field images of zygotes immediately after electroporation at 50 V, 60 V, and 70 V, compared with the untreated control group. **(B)** Representative images of high-quality expanded blastocysts derived from electroporated zygotes cultured *in vitro*. Scale bars, 150 μm.

**Figure S3. Targeted amplicon deep sequencing of *TBXT* and *FecB* gene-edited lambs.** (A) Deep sequencing analysis of the *TBXT* gene-edited lambs. Genomic DNA was extracted from venous blood collected 10 days after birth, and the target region encompassing the editing site was amplified by PCR. The amplicons were subjected to deep sequencing on an Illumina platform. The sequencing reads were aligned to the reference sequence, and the red box indicates the intended cleavage site where insertions or deletions (indels) were detected. (B) Deep sequencing analysis of the *FecB* gene, performed similarly as in (A). The red box highlights the target editing site where mutations were identified.

**Figure S4. Analysis of off-target effects in *TBXT*- and *FecB*-edited lambs.** Potential off-target sites predicted by CRISPOR were PCR-amplified from genomic DNA of edited lambs and sequenced by Nanopore sequencing. **(A)** Sequencing chromatograms of *TBXT* off-target sites. **(B)** Sequencing chromatograms of *FecB* off-target sites. All examined sites displayed single, clear peaks identical to wild-type controls, indicating no detectable off-target editing at the predicted sites.

**Supplementary Tables**

Supplementary Table S1 - epegRNAs sequence

| Gene | epegRNA Name | sequence for spacer, RT, PBS, linker (5'→3') |
| --- | --- | --- |
| *TBXT* | Epeg1 | AACGGGGAGTGGGTGCCGGG, CTGGGGCAAGCCA, GTGGGTGCC, TAAATATC |
| *TBXT* | Epeg2 | AACGGGGAGTGGGTGCCGGG, CTGGGGCAAGCCA, GGAGTGGGTGCC, ATAATAAA |
| *TBXT* | Epeg3 | AACGGGGAGTGGGTGCCGGG, GCTTGCCCCAG, GGCACCCACTCC, GAATAAAT |
| *FecB* | Fepg1 | AGAGACAGAAATATATCAGA, ATCAACACCGTCC, GATATATTTCTGTCTCT, TAAACCTT |
| *FecB* | Fepg2 | TCACTACAGAGGAGGCCAGC, GTCCGATATATTTCTGTCTCTCGGAACCAGCT, GGCCTCCTCTGT, TACATAAT |

The scaffold sequence used for all epegRNAs is

GTTTTAGAGCTAGAAATAGCAAGTTAAAATAAGGCTAGTCCGTTATCAACTTGAAAAAGTGGCACCGAGTCGGTGC The evopreQ1 sequence used for all epegRNAs is CGCGGTTCTATCTAGTTACGCGTTAAACCAACTAGAA

Supplementary Table S2 - Nicking sgRNAs sequence

| Gene | Nicking sgRNA name | sequence for spacer (5'→3') |
| --- | --- | --- |
| *TBXT* | Tn | CCCCGAAGTTGGGGGAGTCG |
| *FecB* | Fn | CAAAAAAAGAGAGGAAAGCT |

Supplementary Table S3- Primers for amplification of the target sites

| Gene | F | R |
| --- | --- | --- |
| *TBXT* | F1: ATCTAGGTGAGTCCTGGGAAGG | R1: GCATATTTTCTCTACCCCAGG |
|  | F2: TGCCTTTGGCCTCACGAT | R2: AAGGAGACAGGTGCCTTCAT |
| *FecB* | F1: tttaccactagcgccacctc | R1: tctgtgaggtctcccattag |
|  | F2: TTTGGATGGGAAAGTGGCGT | R2: AGGAAACCCTGAACATCGCT |

For each target locus, F1/R1 denotes the outer primer pair used for the first-round PCR, whereas F2/R2 denotes the inner primer pair used for the second-round PCR.

Supplementary Table S4 - Primers for *in vitro* transcription

| NO. | F | R |
| --- | --- | --- |
| *TBXT*-Epeg1 | TAATACGACTCACTATAGGGAACGGGGAGTGGGTGC | TTCTAGTTGGTTTAACGCGT |
| *TBXT*-Epeg2 | TAATACGACTCACTATAGGGAACGGGGAGTGGGTGC | TTCTAGTTGGTTTAACGCGT |
| *TBXT*-Epeg3 | TAATACGACTCACTATAGGGAACGGGGAGTGGGTGC | TTCTAGTTGGTTTAACGCGT |
| *FecB*-Fepg1 | TAATACGACTCACTATAGGGAGAGACAGAAATATATC | TTCTAGTTGGTTTAACGCG |
| *FecB*-Fepg2 | TAATACGACTCACTATAGGGTCACTACAGAGGAGGCCA | TTCTAGTTGGTTTAACGCG |
| Tn | TAATACGACTCACTATAGGGCCCCGAAGTTGGGGGAGT | GCACCGACTCGGTGCCACTTTTTC |
| Fn | TAATACGACTCACTATAGGGCAAAAAAAGAGAGGAAAGCT | GCACCGACTCGGTGCCACTTTTTC |

Supplementary Table S5 - Sequences for *in vitro* PE2 nicking and reverse transcription assays.

| Name | Sequence (5’ → 3’) | Length (nt) | Purpose |
| --- | --- | --- | --- |
| dsDNA-FAM -F- | **FAM-**ACCGCTGGAAGTACGTGAACGGGGAGTGGGTGCCGGGGGGCAAGCCAGAGC | 51 | Forward strand of 51-bp *in vitro* substrate |
| dsDNA-R | GCTCTGGCTTGCCCCCCGGCACCCACTCCCCGTTCACGTACTTCCAGCGGT | 51 | Reverse strand of 51-bp *in vitro* substrate |
| pegRNA-spacer | AACGGGGAGTGGGTGCCGGG | 20 | Spacer targeting the 51-bp substrate |
| sgRNA-spacer | AACGGGGAGTGGGTGCCGGG | 20 | Control sgRNA spacer for nicking assay |
| pegRNA-RT1 | GGCTTGCCCCAG | 12 | pegRNA with a 12-nt RT template for *in vitro* extension assay |
| pegRNA-RT2 | CTGGGGCAAGCCA | 13 | pegRNA with a 13-nt RT template for *in vitro* extension assay |

Both pegRNA-RT1 and pegRNA-RT2 share an identical 12-nt PBS (5′-GGCACCCACTCC-3′) and scaffold sequence (provided in Supplementary Table S1).

Supplementary Table S6 - predicted off-target (OT) sites analyzed

| NO. | Off-target site |
| --- | --- |
| *TBXT*-OT1 | TTGCGATGCGCGTACGGGCTGGG |
| *TBXT*-OT2 | AAATTGAAAGTATCCCTTCCAGG |
| *TBXT*-OT3 | CCTTTTCTGACCTAGTCTCAGAA |
| *TBXT*-OT4 | TTGTGATGCCCGCACGTGCTGGG |
| *TBXT*-OT5 | TTCCAAGGCTAGGTCGGAAAAGG |
| *FecB*-OT1 | CCCCATAGCGACCTTTTCCAATC |
| *FecB-*OT2 | CCACATAGCGATCATATCCCATC |
| *FecB*-OT3 | CCCCATATGGACCTCTTCTAATC |
| *FecB*-OT4 | AGTGGGAAAAGGTAGCTATGAG |
| *FecB*-OT5 | TATTGGAAACGGCCGCTTTGGGG |
| *FecB*-OT6 | ATTGGAAAGGCTTGGAATGAGG |
| *FecB*-OT7 | CCCCATAGCAACCTTTCAAAATC |

Supplementary Table S7 - Primers for amplification of predicted off-target (OT) sites

| NO. | F | R |
| --- | --- | --- |
| *TBXT*-OT1 | GGCTCAAGCAGTAGCAGTCA | ACTTTCTGTCCCTCCAGGCAG |
| *TBXT*-OT2 | TAACAGCCCAGAATGCCCTG | TTGTCCCCTTGATGCCTTCC |
| *TBXT*-OT3 | ACAACTTGAGCTGGAGTGTCC | TCCCTCATGATTCCTCATTATGAAG |
| *TBXT*-OT4 | GGCAGCTAAGGACCCACATT | TTCGAATCGCTGGGTTTGGAG |
| *TBXT*-OT5 | GGTGTCTTGGCTAGCTTCCT | ACTCAATGCTTCAACTTCCTCTA |
| *FecB*-OT1 | ACGTGCTCCTTCAAATCCCT | TAGTGCCGTGAACGCACTAAC |
| *FecB*-OT2 | CCAACCCCAAACCTAGCCTT | CTTAAAGGGAAGGCATTGGGGG |
| *FecB*-OT3 | GAAACAGGGAGCCTTTTGGG | AGACTCTCCCTTTCCTGGCTG |
| *FecB*-OT4 | GGAAGCCTAGCTCAGTGTGG | CTCCAAACCCAGCGATTCGAA |
| *FecB*-OT5 | GCTCGCCCCTCTAATCCTTC | CTGTCACCAGCGGACCTTAGG |
| *FecB*-OT6 | AGCTTCCTCCCTCCTTGTCT | CATAGGCATGACCGAGTCCCA |
| *FecB*-OT7 | AGTGCAGCTTTGTAACACTGT | GCTTTTGGAGGAACATCTTGCAA |

Supplementary Table S8 - Embryo development after PE3 RNP electroporation under different voltages

| Voltage (V) | Oocytes (n) | Cleaved embryos (n) | Cleavage rate (Mean ± SEM, %) | Blastocysts (n) | Blastocyst rate (Mean ± SEM, %) |
| --- | --- | --- | --- | --- | --- |
| Control | 197 | 115 | 54.46 ± 9.61^a^ | 23 | 23.05 ± 0.83 |
| 50 | 287 | 148 | 52.04 ± 3.14 ^a^ | 28 | 19.42 ± 1.58 |
| 60 | 256 | 144 | 55.91 ± 1.13 ^a^ | 34 | 21.06 ± 5.92 |
| 70 | 299 | 80 | 26.51 ± 2.06 ^b^ | 11 | 14.43 ± 4.23 |

Electroporation conditions: 3 pulses of 3 ms each. Data are presented as means ± SEM from three independent experiments. Different superscript letters (a, b) within the same column indicate statistically significant differences (P < 0.05).

Supplementary Table S9 - Editing efficiency at different PE2 : pegRNA mass ratios

| PE2:pegRNA mass ratio | Embryos (n) | Edited embryos (n) | Editing efficiency (Mean ± SEM, %) |
| --- | --- | --- | --- |
| 3.5:1 | 156 | 44 | 29.94 ± 10.70 |
| 5:1 | 127 | 29 | 19.97 ± 7.01 |
| 7:1 | 131 | 28 | 19.47 ± 5.81 |
| 14:1 | 126 | 9 | 7.31 ± 2.68 |

Data are presented as means ± SEM from three independent experiments.

Supplementary Table S10 - Editing efficiencies of *TBXT* and *FecB* in the single-target and dual-target co-delivery groups

|  | Editing efficiency in single-target group (No. of edited embryos/ No. of tested embryos) | | | Editing efficiency in dual-target co-delivery group (%, No. of edited embryos/ No. of tested embryos) | | |
| --- | --- | --- | --- | --- | --- | --- |
| *TBXT* editing efficiency(%) | 27.54（19/69） | 20.00（10/50） | 11.76  (2/17) | 11.11  (2/18) | 25.00  (15/60) | 16.67  (5/30) |
| *FecB* editing efficiency(%) | 30.00（6/20） | 17.64  (3/17) | 12.50  (2/16) | 20.00 (6/30) | 15.79  (3/19) | 22.22  (6/27) |

In the dual-target co-delivery group, both *TBXT-* and *FecB-*targeting PE3 RNPs were delivered simultaneously. Each embryo was genotyped at only one locus; therefore, the *TBXT* and *FecB* results were obtained from separate embryo subsets and do not demonstrate co-editing within the same embryo.

Supplementary Table S11 - Developmental competence of zygotes from live donor sheep after PE3 RNP electroporation.
Cleavage and blastocyst rates are shown for electroporated (treated) embryos and untreated controls.

|  | zygotes (n) | Cleaved embryos (n) | Cleavage rate (Mean ± SEM, %) | Blastocysts (n) | Blastocyst rate (Mean ± SEM, %) |
| --- | --- | --- | --- | --- | --- |
| Control | 212 | 157 | 75.57±2.71 | 82 | 52.93±1.92 |
| Electroporated | 198 | 141 | 70.94±2.93 | 81 | 56.70±10.48 |

Supplementary Table S12 - Comparison of tail length and body length at one month of age between *TBXT* gene-edited positive lambs and wild-type controls

| Individual / Group | Tail length (cm) | Body length (cm) |
| --- | --- | --- |
| *TBXT* gene-edited lamb #13 | 15 | 39 |
| *TBXT gene-edited lamb*#07 | 20 | 37 |
| Wild-type control lamb | 27 | 36 |
|  | 24 | 37 |
|  | 21 | 34 |
|  | 30 | 25 |
|  | 25 | 36 |
|  | 26 | 36 |
|  | 25 | 41 |
|  | 24 | 40 |
|  | 22 | 38 |
|  | 24 | 40 |
|  | 21 | 38 |
|  | 25 | 39 |
